# Multiplexed measurement of candidate blood protein biomarkers of heart failure

**DOI:** 10.1002/ehf2.13320

**Published:** 2021-03-28

**Authors:** Claire Tonry, Ken McDonald, Mark Ledwidge, Belinda Hernandez, Nadezhda Glezeva, Cathy Rooney, Brian Morrissey, Stephen R. Pennington, John A. Baugh, Chris J. Watson

**Affiliations:** ^1^ Wellcome‐Wolfson Institute for Experimental Medicine Queen's University Belfast 97 Lisburn Rd Belfast BT9 7BL UK; ^2^ UCD Conway Institute, School of Medicine University College Dublin Dublin Ireland

**Keywords:** Heart failure, Biomarkers, Proteomics, MRM, BNP

## Abstract

**Aims:**

There is a critical need for better biomarkers so that heart failure can be diagnosed at an earlier stage and with greater accuracy. The purpose of this study was to design a robust mass spectrometry (MS)‐based assay for the simultaneous measurement of a panel of 35 candidate protein biomarkers of heart failure, in blood. The overall aim was to evaluate the potential clinical utility of this biomarker panel for prediction of heart failure in a cohort of 500 patients.

**Methods and results:**

Multiple reaction monitoring (MRM) MS assays were designed with Skyline and Spectrum Mill PeptideSelector software and developed using nanoflow reverse phase C18 chromatographic Chip Cube‐based separation, coupled to a 6460 triple quadrupole mass spectrometer. Optimized MRM assays were applied, in a sample‐blinded manner, to serum samples from a cohort of 500 patients with heart failure and non‐heart failure (non‐HF) controls who had cardiovascular risk factors. Both heart failure with reduced ejection fraction (HFrEF) patients and heart failure with preserved ejection fraction (HFpEF) patients were included in the study. Peptides for the Apolipoprotein AI (APOA1) protein were the most significantly differentially expressed between non‐HF and heart failure patients (*P* = 0.013 and *P* = 0.046). Four proteins were significantly differentially expressed between non‐HF and the specific subtypes of HF (HFrEF and HFpEF); Leucine‐rich‐alpha‐2‐glycoprotein (LRG1, *P* < 0.001), zinc‐alpha‐2‐glycoprotein (*P* = 0.005), serum paraoxanse/arylesterase (*P* = 0.013), and APOA1 (*P* = 0.038). A statistical model found that combined measurements of the candidate biomarkers in addition to BNP were capable of correctly predicting heart failure with 83.17% accuracy and an area under the curve (AUC) of 0.90. This was a notable improvement on predictive capacity of BNP measurements alone, which achieved 77.1% accuracy and an AUC of 0.86 (*P* = 0.005). The protein peptides for LRG1, which contributed most significantly to model performance, were significantly associated with future new onset HF in the non‐HF cohort [Peptide 1: odds ratio (OR) 2.345 95% confidence interval (CI) (1.456–3.775) *P* = 0.000; peptide 2: OR 2.264 95% CI (1.422–3.605), *P* = 0.001].

**Conclusions:**

This study has highlighted a number of promising candidate biomarkers for (i) diagnosis of heart failure and subtypes of heart failure and (ii) prediction of future new onset heart failure in patients with cardiovascular risk factors. Furthermore, this study demonstrates that multiplexed measurement of a combined biomarker signature that includes BNP is a more accurate predictor of heart failure than BNP alone.

## Introduction

Heart failure (HF) is a major global health issue, with recent estimates suggesting that there are more than 26 million HF patients worldwide. The burden of this disease on the healthcare system is significant. In the United Kingdom, 1–2% of the National Health Service's budget is spent on HF, with 60–70% of this estimated to be on the cost of hospitalizations.^1^ HF is a complex pathology and often presents with non‐specific symptoms. Hence, delays in accurate diagnosis and appropriate treatment interventions contribute further to HF‐associated healthcare costs.^2^ Echocardiography remains key to accurate diagnosis of HF; however, the resources required for this is an important factor in the healthcare burden of HF.^1^ Blood‐based biomarkers offer a lower cost, minimally invasive alternative for HF diagnosis and prognosis, with much quicker turnaround times for test results. The current gold‐standard biomarkers for HF are the natriuretic peptides—N‐terminal proBNP and BNP. However, there are a number of limitations with these biomarkers in the context of HF management; the biological variation of BNP or N‐terminal proBNP is ~30% in chronic HF, and levels are further influenced by patient weight, comorbidities, and medications.^2^ The natriuretic peptides are also not useful in classifying types of HF, which is key to managing the disease effectively—especially in terms of risk stratification. Ultimately, follow‐up echocardiography is still relied upon to confirm diagnosis of HF, even if elevated levels of BNP are detected. The underlying mechanisms that contribute to HF remain poorly understood, and it is thought that biomarkers that reflect important pathophysiologic pathways involved in cardiovascular dysfunction would likely be of greater clinical value for diagnosis and prognosis of HF.

Recent studies indicate that shifting the focus from conventional risk factors and/or single disease biomarkers toward biomarker ‘signatures’, made up of multiple disease‐relevant proteins, would be of considerable benefit in the management of HF.^3^ Aside from cancer, cardiovascular research is the field in which novel disease biomarkers have been most extensively investigated, with more than 150 potential biomarkers for cardiovascular disease documented in the literature.^4^ However, no new protein biomarker tests for HF have yet been approved and included in guidelines for use in clinical practice. This is largely due to difficulties in being able to demonstrate that use of novel biomarkers will eventually lead to improved outcomes for HF patients. Some of the difficulty lies in evaluating lengthy lists of candidate biomarker proteins in a statistically relevant number of patient samples, with sufficient sensitivity and specificity toward HF.^5^ This bottleneck has previously been attributed to the fact that the technologies being used are unable to provide the combination of sufficient throughput and robust accuracy for analysing multiple biomarker candidates in large patient cohorts. In contrast to the traditional antibody‐based assays, mass spectrometry technology allows for more high‐throughput, sensitive, and selective measurement of target proteins by detection of their unique peptide fragments. Multiple reaction monitoring (MRM) is a mass spectrometry technique that makes use of multiple mass analysis steps to select a series of predefined ions for detection.^6^ MRM is advantageous over alternative biomarker validation techniques such as enzyme‐linked immunosorbent assay, SOMA scan and proximity extension assay, in that it is not array‐based and restrictive to measurement of protein targets to which suitable antibodies or somamers are commercially available. MRM assays are customizable, high‐throughput, and suitable for routine use in a clinical setting.^7^

The key aim of this study was to develop a set of robust MRM assays for measurement of a panel of pathophysiologically relevant candidate protein biomarkers for HF. With the ultimate aim of being able to develop a clinically useful biomarker test, the assays were optimized for application to crude patient serum samples. Application of these assays to a statistically powered patient cohort has provided evidence that this biomarker panel could have clinical utility for prediction of HF within a mixed population of both disease and control patients.

## Methods

Full detailed methods are available in the supporting information.

### Study population

Patients for this study were recruited from the HF Unit and the Blood Pressure Unit at St. Michael's Hospital in Dun Laoghaire, Co. Dublin. The Ethics Committee at St. Vincent's University Hospital approved the study protocol, which conformed to the principles of the Helsinki Declaration.

### Serum collection and preparation for multiple reaction monitoring analysis

Peripheral venous blood samples were obtained during clinical assessment. Point of care BNP was measured using a Triage meter (Biosite). Crude and depleted samples were digested with trypsin and de‐salted using C18 resin ZipTips® (Millipore).

### Multiple reaction monitoring assay design and data analysis

Multiple reaction monitoring analysis was carried out with nanoflow reverse phase C18 chromatographic Chip Cube‐based separation coupled to an Agilent 6460 triple quadrupole mass spectrometer. Skyline (MacCoss laboratory, Washington DC version 1.4), Spectrum Mill Peptide Selector (Agilent Technologies, version 3.3.078), and Qualitative Mass Hunter Software (Agilent, V 3.3.078) were used for MRM assay design and data analysis. IBM SPSS Statistics Version 24 was used for statistical analysis. Non‐parametric Mann–Whitney U and Kruskal–Wallis tests were used to investigate differences in biomarker levels and continuous clinical variables between groups. A one‐way analysis of covariance (ANCOVA) was conducted to compare biomarker expression between groups, while controlling for confounding clinical factors. Survival analysis was performed using the Cox‐regression method. Random Forest models were used to discriminate between the patient groups (Random Forest package in R V.4.3.2 and pROC package in R V.3.4.4).

## Results

### Multiple reaction monitoring assay development—selection of proteotypic peptides

A panel of 35 candidate biomarker proteins relevant to cardiovascular conditions was assembled. This panel included 19 known and 16 novel biomarkers. ‘Known’ biomarkers were identified from the literature and added to the panel based on their known involvement in the pathogenesis of cardiovascular diseases (*Table* *1*). ‘Novel’ biomarkers were identified from a previous study by Watson *et al*.^8^ Both Skyline and Peptide Selector were used to select proteotypic peptides for development of MRM assays for the candidate protein biomarkers. Data from in house experiments and relevant publications on MRM‐based investigations also guided the peptide selection process^9^, ^10^, ^11^, ^12^, ^13^, ^14^ (Supporting Information, *Table* S1). Where possible, at least two peptides were selected per protein. It was found that the peptides identified for natriuretic peptide receptor A (ANP) and BNP were not unique to these proteins so they were removed from the candidate list.

**Table 1:** Patient demographic table

Variable	Non‐HF	HFPEF	HFREF	*P* value
	*n* = 287	*n* = 57	*n* = 62	
Age (years)	67.04 ± 9.6	74.19 ± 6.9	70.12 ± 11.4	0.000*, ^Ŧ^
Male (%)	44.90% (*n* = 129)	63.20% (*n* = 36)	72.60% (*n* = 45)	0.000
SBP (mmHg)	135.20 ± 18.0 (*n* = 283)	121.96 ± 18.9	116.39 ± 21.9	0.000*, ^Ŧ^
DBP (mmHg)	81.88 ± 11.0 (*n* = 283)	74.47 ± 12.5	69.15 ± 11.0	0.000*, ^Ŧ^, ^¥^
Heart rate (bpm)	68.49 ± 12.4 (*n* = 283)	69.78 ± 13.4 (*n* = 55)	66.97 ± 13.7	0.257
Body mass index (kg/m^2^)	28.51 ± 5.0	31.62 ± 5.9 (*n* = 56)	27.69 ± 4.1	0.001^Ŧ^, ^¥^
BNP (pg/mL)	40.93 ± 47.0	280.40 ± 218.4	200.60 ± 189.1	0.000*, ^Ŧ^
**Medical history**				
Cardiac failure	0.30%	100%	100%	0.000
Arr/Atrial fibrillation	14.30%	85.50%	56.50%	0.000
Coronary artery disease/ischemic heart disease	18.50%	41.10% (*n* = 56)	59.70%	0.000
LipD	77.40% (*n* = 222)	61.40% (*n* = 35)	71.00% (*n* = 44)	0.525
Diabetes mellitus	17.10% (*n* = 49)	29.80% (*n* = 17)	15.30% (*n* = 11)	0.013
Stroke	3.50% (*n* = 10)	8.60% (*n* = 35)	3.30% (*n* = 61)	0.144
Hypertension	74.60%	86.00%	32.30% (*n* = 62)	0.000
Angina	6.60%	11.40% (*n* = 35)	16.00%	0.072
Arthritis	22.60%	25.00% (*n* = 20)	21.30% (*n* = 61)	0.128
COPD	1.70%	11.40% (*n* = 35)	16.10%	0.000
Asthma	8.40%	9.10% (*n* = 35)	6.50%	0.460
Cancer	6.30%	15.00% (*n* = 20)	16.80%	0.001
**Medications**				
ACEi	27.90%	68.40%	64.50%	0.000
ARB	32.10%	15.00% (*n* = 20)	24.20%	0.155
Spironolactone	0.30%	0.00% (*n* = 42)	4.80%	0.005
Beta blocker	29.30%	14.00%	82.30%	0.000
Calcium channel blocker	28.20%	28.10%	4.80%	0.000
Statin	70.00%	59.60%	62.90%	0.219
Alpha blocker	8.70%	11.90% (*n* = 42)	3.40% (*n* = 58)	0.028
Aspirin	43.20%	50.90%	56.50%	0.125
Loop diuretics	5.60%	94.70%	71.00%	0.000
Warfarin	6.60%	68.40%	37.10%	0.000
Dig 1	1.40%	30.00% (*n* = 20)	24.20%	0.000
Insulin	1.70%	4.80% (*n* = 42)	1.60%	0.420
Nitrate	4.20%	45.00% (*n* = 20)	17.70%	0.000
Ivabradine	0.70%	0.00% (*n* = 20)	4.80%	0.033
Clopidogrel	3.80%	5.00% (*n* = 20)	8.10%	0.353
**Bloods**				
High density lipoprotein	1.37 ± 0.5 (*n* = 286)	1.10 ± 0.4 (*n* = 24)	1.16 ± 0.4 (*n* = 61)	0.000*, ^Ŧ^
Low density lipoprotein	2.68 ± 0.9 (*n* = 261)	3.11 ± 5.1 (*n* = 22)	2.28 ± 0.8 (*n* = 53)	0.000*, ^Ŧ^
Cholesterol	4.80 ± 1.0	4.09 ± 0.9 (*n* = 50)	4.18 ± 1.1	0.000*, ^Ŧ^
Triglyceride	1.70 ± 1.0 (*n* = 286)	1.56 ± 0.7 (*n* = 50)	1.85 ± 1.3	0.945
**Echocardiography**				
Ejection fraction (%)	67.09 ± 8.6	61.66 ± 7.4 (*n* = 56)	40.70 ± 15.1 (*n* = 57)	0.000*, ^Ŧ^, ^¥^
LVIDd (mm)	46.22 ± 5.5	49.50 ± 5.2 (*n* = 52)	57.78 ± 10.7 (*n* = 54)	0.000*, ^Ŧ^, ^¥^
IVSd (mm)	11.25 ± 1.9	12.48 ± 2.6 (*n* = 54)	11.30 ± 3.3 (*n* = 50)	0.005*
PWd (mm)	9.68 ± 1.6	10.72 ± 1.9 (*n* = 54)	9.63 ± 2.3 (*n* = 52)	0.001*, ^¥^
LV mass (gm)	174.57 ± 55.1	222.69 ± 60.64 (*n* = 52)	239.19 ± 89.4 (*n* = 52)	0.000*, ^Ŧ^
LVMI (gm/m^2^)	91.98 ± 24.2	112.44 ± 30.4 (*n* = 53)	127.14 ± 40.4 (*n* = 48)	0.000*, ^Ŧ^
LA volume (mL)	51.59 ± 19.1 (*n* = 275)	105.20 ± 44.2 (*n* = 44)	84.48 ± 23.5 (*n* = 33)	0.000*, ^Ŧ^
LAVI (mL/m^2^)	26.75 ± 9.1	53.33 ± 20.3 (*n* = 46)	44.04 ± 11.07 (*n* = 32)	0.000*, ^Ŧ^
e' (cm/s)	8.20 ± 2.5 (*n* = 284)	9.50 ± 2.2 (*n* = 45)	7.80 ± 3.5 (*n* = 44)	0.001*, ^¥^
E/e' ratio	9.00 ± 2.9 (*n* = 282)	11.29 ± 3.9 (*n* = 46)	9.79 ± 4.9 (*n* = 43)	0.001*, ^¥^
LA Dimen (mm)	37.91 ± 6.0 (*n* = 285)	49.33 ± 6.2 (*n* = 52)	44.81 ± 7.0 (*n* = 54)	0.000*, ^Ŧ^, ^¥^

ACEi, angiotensin converting enzyme inhibitor; ARB, angiotensin receptor blocker; Arr, arrhythmia; COPD, chronic obstructive pulmonary disease; e', mitral flow velocity; E/A, ratio of maximal early to late (atrial) transmitral filling velocities in diastole; E/e', ratio of mitral early diastolic flow velocity over tissue Doppler lateral mitral annular lengthening velocity; g/dL, grams per decilitre; IVSd, intraventricular septum in diastole; LA, left atrial; LAVI, left atrial volume; LipD, dyslipidemia; LV, left ventricular; LVIDd/LVIDs, left ventricular diastolic/systolic dimensions; LVMI, left ventricular mass; MI, myocardial infarction; PWd, posterior wall in diastole; SBP/DBP, systolic/diastolic blood pressure.

Values are mean ± SD, *n* (%). Independent‐samples Kruskal–Wallis analysis was performed for numerical variables for the three patient groups. Chi‐square contingency analysis was performed for categorical variables.

* *P* values that indicate significant differences (*P* < 0.05) between non‐HF and HFPEF group.

^Ŧ^ *P* values that indicate significant differences (*P* < 0.05) between non‐HF and HFREF group.

^¥^ *P* values that indicate significant differences (*P* < 0.05) between HFPEF and HFREF group.

### Multiple reaction monitoring development—assay optimization

**Figure 1:**
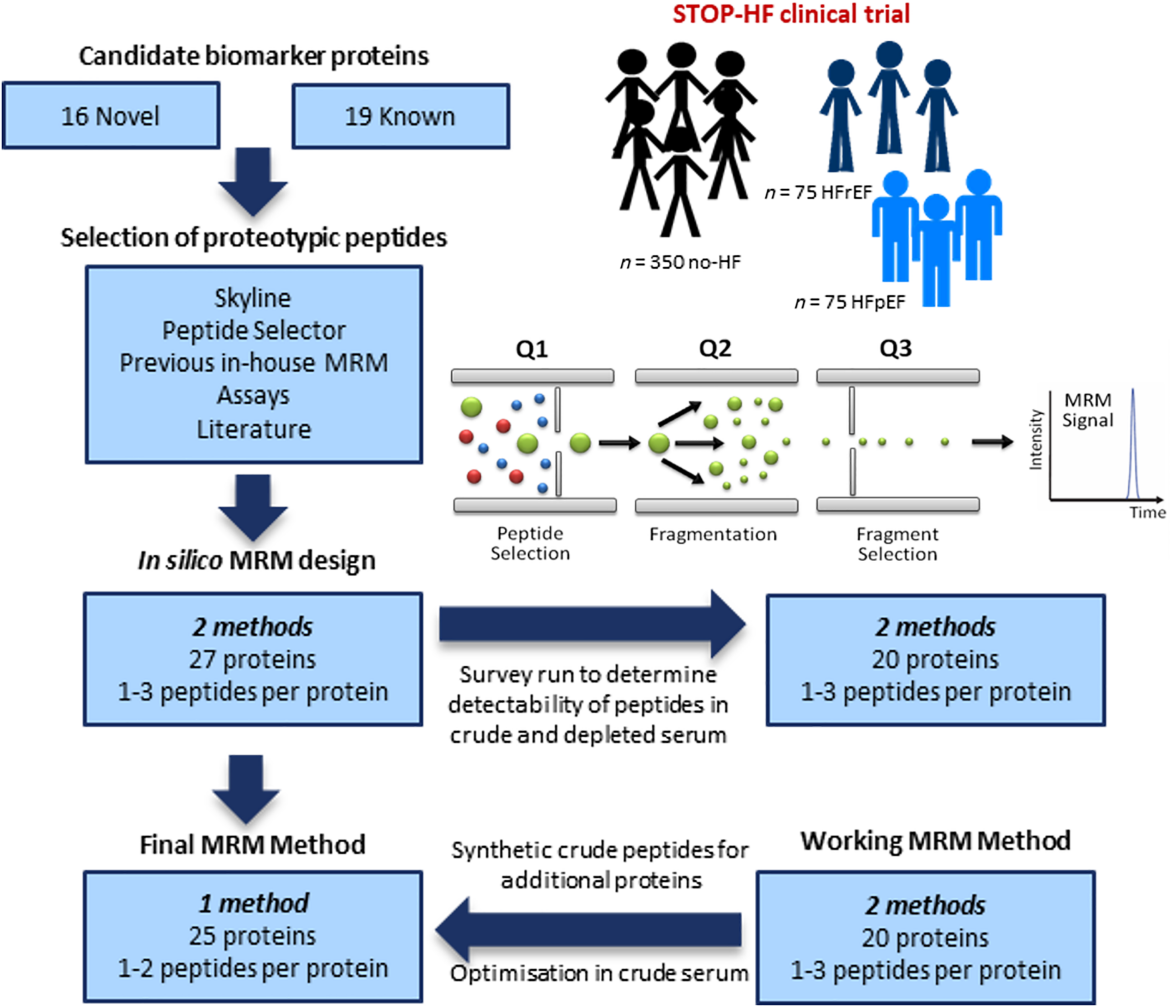
Workflow for MRM assay development and optimisation. The figure illustrates the process of MRM assay design and optimisation. Proteotypic peptides were carefully selected using Skyline and Peptide Selector software as well as data from previous in‐house MRM‐based studies and relevant publications. MRM assays were initially developed in depleted serum and further optimized in crude serum. Synthetic peptides were used to optimize parameters for low abundant peptide targets. Working assays were ultimately compiled into a single MRM method for measurement of 25 proteins. MRM, multiple reaction monitoring.

The *in‐silico* MRM assays were analysed in pooled patient serum samples. Crude serum was depleted of the 14 most abundant serum proteins to enhance detectability and optimize measurement parameters for the lower abundant proteins. On the basis of the results from these experiments, MRM assays were optimized for 20 proteins (30 peptides and 150 transitions). These assays were evaluated in crude serum samples in order to confirm that target proteins could be reliably measured without need for serum depletion. This resulted in the development of a single working MRM method for measurement of 22 peptides (15 proteins) (*Figure* S1). Synthetic crude peptides were used to assist in the development of assays for 11 of the remaining proteins, which were determined—based on previous results—to have the most potential for successful measurement in serum and were also considered to be the most biologically relevant. All peptides selected for inclusion in the final MRM method had dot products greater than 0.9. For many of the proteins, only one peptide was included in the final method. To ensure accurate peptide detection, especially for the lower abundant proteins, all five transitions for each peptide were retained in the final method. The resulting final MRM method consisted of 35 peptides for 25 proteins, with a total of 175 transitions. The workflow for peptide selection and assay optimisation is outlined in *Figure* *1*.

### Clinical cohort for assay evaluation

The clinical cohort included 150 HF patients; 75 HF patients with reduced ejection fraction (HFrEF) and 75 HF patients with preserved ejection fraction (HFpEF). A population of 350 non‐heart failure (non‐HF) patients were selected from the STOP‐HF (St. Vincent's Screening to Prevent HF) study. These patients represent a high‐risk population for future development of HF and served as a control group within this study.^15^ Point of care BNP measurements were taken for each patient. Assays, with sensitivity for BNP at <5 ng/mL, were run singly and the intra‐assay variability was <10%.

All samples were analysed singly with the developed MRM method over 21 batches of between 22 and 24 samples. A stock peptide mix was used to confirm system suitability and ensure reproducibility between each batch of samples analysed. The %CV values for the stock peptide mix was around 15% indicating that instrument performance was consistent throughout the analysis. However, it was noted from inspection of the quality of peptide peaks in Skyline, that some batches had a disproportionate amount of missing values or very low values for all peptides. These sample batches were removed from subsequent analyses to remove any potential bias of batch effects. Biomarker analysis was ultimately conducted using data from 406 patient samples (*Table* S2). Even though samples were removed from the analyses, the ratio of HF to non‐HF patients remained the same; 121 HF and 289 non‐HF. Patient characteristics for the 406 patients are summarized in *Table* *1*. The non‐HF patients were generally younger (66.75 ± 96) than HFpEF (74.19 ± 6.9) and HFrEF (70.12 ± 11.4) patients (*P* ≤ 0.001). The majority of patients affected by either HFpEF or HFrEF were male in this cohort (63.2% and 72.6%, respectively). This is unsurprising as, although more women die from HF, it is predominantly diagnosed in men. As indicated in this table, the non‐HF group demonstrate clinical features, which would indicate that they are at risk of future development of HF (prevalence of hypertension = 66.6% and prevalence of dyslipidaemia = 67.6%); however, previous incidences of ischaemic heart disease (IHD, 17.4%) and cardiac arrhythmia/atrial fibrillation (AF, 10.1%) are significantly lower than in the HFpEF and HFrEF groups (IHD = 41.1%, AF = 85.5% vs. IHD = 59.7%, AF = 56.5%, respectively, *P* ≤ 0.001). Within the HF group, HFpEF patients demonstrate overall poorer cardiovascular health compared with HFrEF, with higher body mass index (31.72 ± 5.9 vs. 27.69 ± 4.1, *P* = 0.001), and higher incidence of diabetes (29.8% vs. 15.3%, *P* = 0.001), stroke (8.6% vs. 3.3%, *P* = 0.144), and hypertension (86.0% vs. 32/3%, *P* ≤ 0.001).

### Evaluation of individual candidate biomarkers

Area values for the most intense peptide transition were used to determine peptide expression levels in sera. Any missing values were replaced using a multiple imputation method to remove any potential bias. As expected, BNP was significantly differentially expressed between non‐HF and HF patients; however, there was not a significant difference in BNP expression between HFrEF and HFpEF patients once data was adjusted for AF (*P* = 0.320, *Figure S2*). This highlights the lack of specificity of BNP for differentiation between types of HF , which is required for appropriate clinical management of HF. Individually, five of the candidate biomarker proteins were significantly differentially expressed between HF and non‐HF patients: leucine‐rich‐alpha‐2‐glycoprotein (LRG1, *P* < 0.001), zinc‐alpha‐2‐glycoprotein (ZA2G, *P* = 0.001), serum paraoxanse/arylesterase (PON1, *P* = 0.006), Apolipoprotein A‐I (APOA1, *P* = 0.009 and *P* = 0.038), and pentraxin 3 (PTX) (*Table* *2*, *P* = 0.049). In addition to gender and age, adjustments for AF were made as a large proportion (82%) of patients within this cohort had the condition. All proteins remained significant when adjusted for gender, age and AF individually, aside from PTX, which was no longer significant after adjustment for these confounders (*Table* *2*). Only peptides for APOA1 protein remained significant when controlled for all three confounders collectively (*P* = 0.013 and *P* = 0.046). Four proteins were significantly differentially expressed between non‐HF and the subtypes of HF (HFrEF and HFpEF): LRG1 *(P* < 0.001), ZA2G (*P* = 0.005), PON1 (*P* = 0.013), and APOA1 (*P* = 0.038). LRG1 was significantly differentially expressed between non‐HF and HFpEF, and this held true when adjusted for gender and age (*P* = 0.001 and *P* = 0.040, respectively). However, significance was lost when adjusted for AF or all three confounders collectively (*Table* *3*). ZA2G was significantly differentially expressed between non‐HF and HFrEF and also between non‐HF and HFpEF (*P* = 0.005 and *P* = 0.043, respectively). Differences between non‐HF and HFrEF remained significant when adjusted for age (*P* = 0.025) and AF (*P* = 0.031) but not when adjusted for gender or all three confounders collectively (*Table* *3*). PON1 was significantly differentially expressed between non‐HF and HFrEF (*P* = 0.005). Significance remained when adjusted for age, gender, and AF individually and all three confounders collectively (*P* = 0.05). APOA1 was significantly differentially expressed between non‐HF and HFrEF (*P* = 0.038) and remained significant when adjusted for AF (*P* = 0.048) but not when adjusted for any other confounders (*Table* *3*).

**Table 2:** Significantly differentially expressed proteins HF vs. non‐HF

				Adjust for gender	Adjust for Afib	Adjust for Age	Adjust for all three
Protein	Mean rank noHF	Mean rank HF	*P* value	*P* value	*P* value	*P* value	*P* value
Leucine‐rich alpha‐2‐glycoprotein	191.8	238.3	0.000***	0.000***	0.048*	0.002**	0.100
Zinc alpha‐2‐glycoprotein	193.5	234.3	0.001***	0.014*	0.010**	0.006**	0.100
Serum paraoxanase/arylesterase	216.1	180.3	0.006**	0.008**	0.020*	0.021*	0.100
Apolipoprotein A‐I^a^	215.5	181.7	0.009**	0.024*	0.005**	0.010**	0.013*
Apolipoprotein A‐I^a^	213.4	186.7	0.038*	0.100	0.035*	0.100	0.046*
Pentraxin 3	197.1	225.7	0.049*	0.200	0.100	0.100	0.200

HF, heart failure.

^a^ Repetition of Apolipoprotein A‐I reflective of the two different peptides measured for this protein.

* *P* ≤ 0.05.

** *P* ≤ 0.01.

*** *P* ≤ 0.001.

**Table 3:** Significant protein changes in HF subtypes

				Pairwise comparisons														
				Unadjusted *P* value			Adjust for gender *P* value^a^			Adjust for Afib *P* value^a^			Adjust for age^a^			Adjust for all three^a^		
Peptide	Mean rank non‐ HF	Mean rank HFrEF	Mean rank HFpEF	NoHF vs. HFrEF	NoHF vs. HFpEF	HFrEF vs. HFpEF	NoHF vs. HFrEF	NoHF vs. HFpEF	HFrEF vs. HFpEF	NoHF vs. HFrEF	NoHF vs. HFpEF	HFrEF vs. HFpEF	NoHF vs. HFrEF	NoHF vs. HFpEF	HFrEF vs. HFpEF	NoHF vs. HFrEF	NoHF vs. HFpEF	HFrEF vs. HFpEF
Leucine‐rich alpha‐2‐glycoprotein	191.78	222.29	255.05	0.06	0.00***	0.11	0.20	0.00***	0.40	0.71	0.07	0.74	0.29	0.04*	0.56	0.70	0.11	0.93
Zinc alpha‐2‐glycoprotein	193.45	240.42	227.84	0.01**	0.04*	0.57	0.08	0.34	1.00	0.03*	0.35	1.00	0.03*	0.37	1.00	0.15	0.96	1.00
Serum paraoxanase/arylesterase	188.44	149.67	168.14	0.01**	0.17	0.76	0.02*	0.49	0.85	0.03*	0.72	0.87	0.02*	0.81	0.66	0.05	0.91	0.78
Apolipoprotein A‐I	215.46	180.79	182.69	0.04*	0.05	0.89	0.25	0.24	1.00	0.05*	0.07	1.00	0.11	0.18	1.00	0.12	0.12	1.00

Afib, atrial fibrillation; APOA1, Apolipoprotein A1; HF, heart failure; LRG1, leucine‐rich‐2‐glycoprotein; PON1, serum paraoxanase/arylesterase; ZA2G, zinc‐alpha‐2‐glycoprotein.

^a^ Corrected *P* value refers to Bonferroni correction applied for analysis of variance and analysis of covariance.

* *P* ≤ 0.05.

** *P* ≤ 0.01.

*** *P* ≤ 0.001.

### Predictive capacity of combined biomarkers

**Figure 2:**
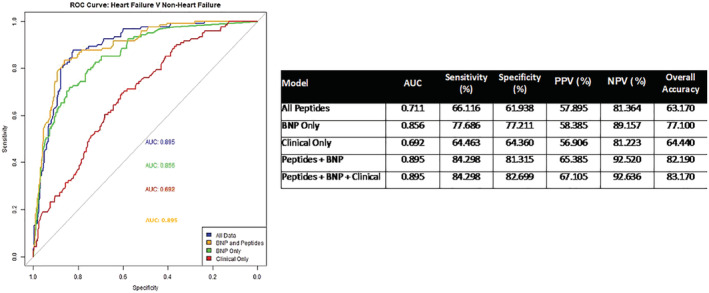
Performance of biomarkers and BNP in prediction of heart failure. (A) Receiver operating curve demonstrating predicate value of BNP alone (green), clinical information alone (red), BNP combined with peptides (amber), and BNP combined with peptides and clinical information (blue). ‘Clinical’ information refers risk factors: patient age, sex, and body mass index. AUC, area under the curve.

A random forest model was developed to determine if collective measurement of all 25 proteins improves accuracy of BNP for prediction of HF. This type of modelling is designed to handle artefactual noise, and thus, all peptides can be included in the model without diminishing the validity of statistical interpretations from the model, that is, variable detectability of some of the lower abundant peptides did not affect model performance.^16^ Indeed, removing peptides that had only a modest contribution to the model, resulted in poorer model performance (data not shown). The model developed here was shown to predict HF with an accuracy of 83.17% and area under the curve (AUC) of 0.90 (*Figure* *2*). In contrast, a model, which only included patient BNP data, had 77.1% accuracy and an AUC of 0.86. Contribution of each individual protein to the model performance is outlined in *Table* *4*, where proteins are listed in order of importance to the model's predictive capacity. The performance of the model was not enhanced any further with addition of risk factors such as patient age, sex, and body mass index, and these risk factors alone achieved accuracy of just 64.4% and an AUC of 0.69 (*Figure* *2*). A one‐sided hypothesis test was carried out based on 2000 stratified bootstrap samples to test if the AUC obtained using only BNP was significantly less than the AUC achieved with addition of peptide measurements. It was found that the difference was significant (*P* = 0.0045). The net reclassification was also calculated [net reclassification 0.1111 95% confidence interval (CI) (0.0209–0.2013) *P =* 0.01578); however, results varied based on which cut‐off threshold values were chosen. Hence, the bootstrapping approach provided more stable results for comparison of model performance. Age is a confounding factor in the prediction of HF. Logistic regression analysis was also performed on a subset of age‐matched HF and non‐HF patients (*n* = 95 vs. *n* = 208, respectively) to ensure that the overall model performance was not influenced by effects of age. It was found that overall predictive performance of all peptides combined remained similar (0.83) to the non‐matched cohort, as determined by observations of AUC (data not shown).

**Table 4:** Proteins included in predictive model for heart failure vs. non‐heart failure in order of importance

Protein^a^	Importance score
Leucine‐rich‐alpha 2 glycoprotein	7.684
Transthyretin	5.263
Zinc‐alpha‐2‐glycoprotein	4.817
Apolipoprotein A‐I	4.728
Leucine‐rich‐alpha 2 glycoprotein	4.165
Apolipoprotein A‐I	4.119
Serum paraoxonase/arylesterase 1	3.865
Pigment epithelium‐derived factor	3.859
Complement C3	3.559
Fibronectin	3.412
Apolipoprotein A‐IV	3.338
Complement factor I	3.289
Beta‐2‐glycoprotein	3.241
Pentraxin‐related protein PTX3	3.195
Metalloproteinase inhibitor 1	3.195
Beta‐2‐glycoprotein	3.124
Apolipoprotein A‐IV	3.079
Collagen alpha‐1(III) chain	3.062
Vitamin D‐binding protein	3.047
Macrophage migration inhibitory factor	3.020
Complement C3	3.016
Complement C1s subcomponent	3.013
72 kDa type IV collagenase	2.983
Matrix metalloproteinase‐9	2.926
Serum amyloid P‐component (SAP)	2.821
Clusterin	2.764
Gelsolin	2.739
Collagen alpha‐2(I) chain	2.728
Complement factor I	2.717
Collagen alpha‐2(I) chain	2.708
Gelsolin	2.559
Galectin‐3	2.546
Pentraxin‐related protein PTX3	2.147
Interleukin‐6	2.123
Galectin‐3	2.017

^a^ More than one peptide was measured per protein.

### Prediction of future heart failure

**Figure 3:**
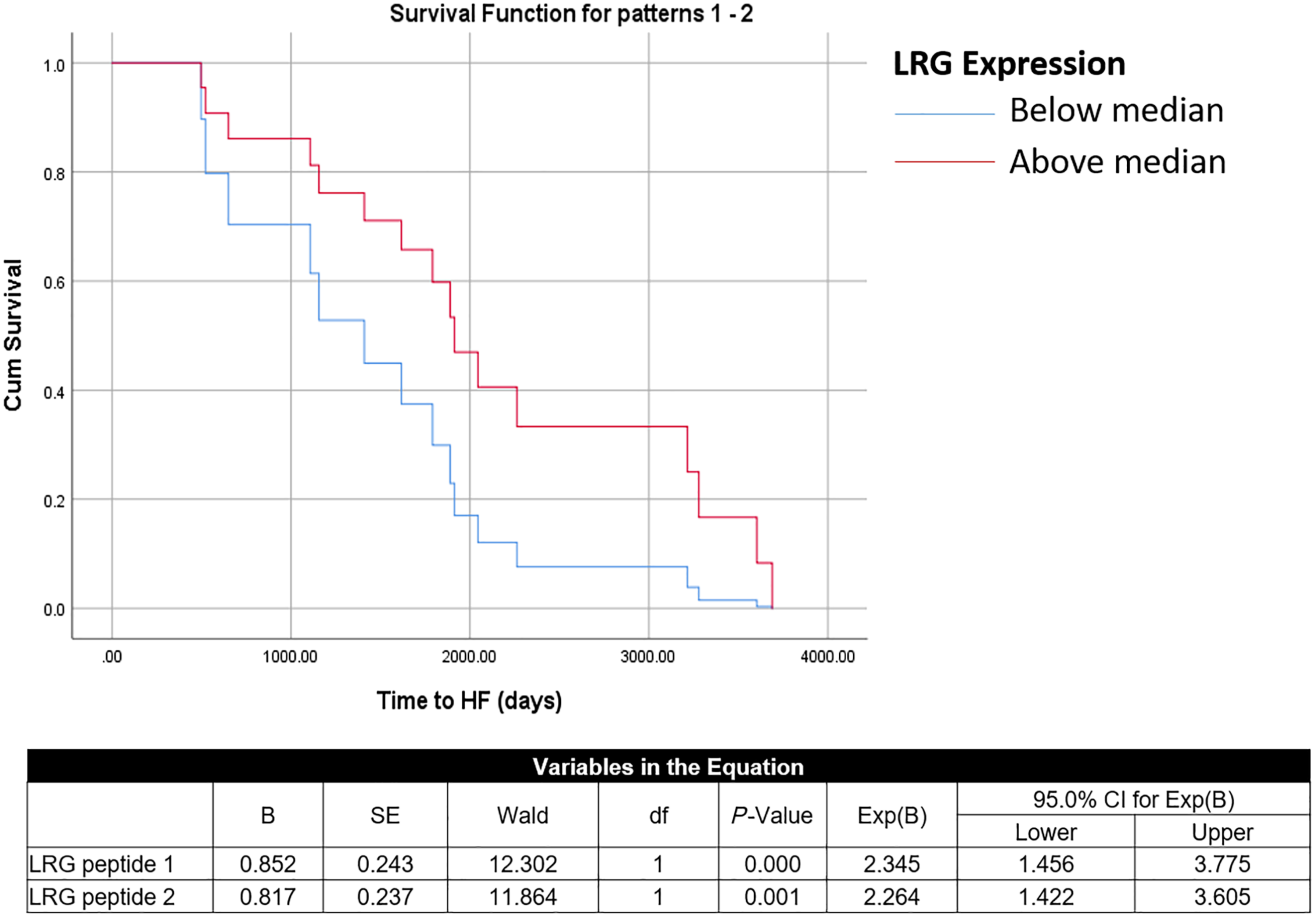
Survival analysis of patients with low and high LRG‐1 protein expression at baseline. CI, confidence interval.

Over a period of 10 years following original sample collection, 17 out of the 287 non‐HF patients developed HF. One of the protein biomarkers, LRG1, was found to be significantly associated with future HF, even when adjusted for age and gender (*Table* *5*). Moreover, it was observed that patients in a ‘high LRG1’ group (with median expression used as the cut‐off) were twice as likely to develop HF in the future, and within a shorter amount of time [peptide 1: odds ratio (OR) 2.345 95%CI (1.456–3.775) *P* = 0.000; peptide 2: OR 2.264 95% CI (1.422–3.605), *P* = 0.001] (*Figure* *3*).

**Table 5:** Proteins associated with future HF

				Adjusted for age	Adjusted for gender	Adjusted for Afib	Adjusted for all
Protein	NoHF (*n* = 272)	HF (*n* = 17)	*P* value	*P* value	*P* value	*P* value	*P* value
Leucine‐rich alpha‐2‐glycoprotein (Peptide 2)	3209.10	4393.10	0.042*	0.060	0.040*	0.086	0.077
Leucine‐rich alpha‐2‐glycoprotein (Peptide 2)	2477.64	3385.53	0.026*	0.045*	0.030*	0.055	0.077

Afib, atrial fibrillation; HF, heart failure.

* *P* ≤ 0.05.

## Discussion

In this study, an MRM‐mass spectrometry approach was employed for multiplexed measurement of 25 candidate protein biomarkers for HF. The ‘novel candidates’ were identified in a previous study by Watson *et al*, in which 2D‐DIGE proteomics analysis of coronary sinus blood revealed differences in protein expression between asymptomatic hypertensive patients, stratified according to BNP levels. In that study, only the protein LRG1 was further validated via enzyme‐linked immunosorbent assay, whereas here, we were able to collectively measure all identified proteins via MRM as part of a biomarker ‘signature’.^8^ For implementation into clinical use, biomarker signatures must bring significant added value to what is already used routinely in the clinic. Hence, a random forest algorithm was used to test the predictive utility of the biomarker signature in combination with BNP. Random forest has desirable properties in that it does not over‐fit the data and can informally allow the assessment of complex high‐order interactions.^17^ This analysis revealed that, by combining measurement of a panel of disease relevant proteins with BNP measurements in a predictive model for HF, the accuracy increases from 77.1% (AUC 0.86) for BNP alone to 83.17% (AUC 0.90) for BNP plus biomarkers. Importantly, the positive predictive value also increases from 58.4% to 65.4%. The majority of peptides were significantly correlated with each other, although the strength of this association was very weak; only peptides associated with the same protein had strong correlations with each other (*r* ≥ 0.8, *Table* S3). LRG, ZA2G, and PON1 were the only proteins to show a significant correlation with BNP, but again these correlations were weak (*r* < 0.4) (*Table* S3).

Individually, a number of the protein candidates show significant differences in expression between HF patients and non‐HF controls and were found to be associated with particular HF aetiologies, that is, HFrEF or HFpEF. This is of interest as, due to poorly understood differences in pathophysiology between HFpEF and HFrEF, many patients with HFpEF are not diagnosed correctly using conventional biomarkers for HF.^18^ Indeed, from the information provided in *Table* *1*, it is evident that the majority of measurements taken from the blood and urine do not discriminate between HFpEF and HFrEF. LRG1 was significantly elevated in HF and particularly associated with HFpEF. Indeed, LRG1 was the only biomarker candidate to show a significant association with HFpEF as opposed to HFrEF, even when adjusted for gender and AF (*Table* *3*). This is noteworthy considering the challenges in diagnosing HFpEF. LRG1 expression was also significantly correlated with BNP expression (*Table* S3). Watson *et al* have previously reported overexpression of LRG1 in asymptomatic patients with elevated BNP, who are at risk for HF. Indeed, there is growing evidence to support the functional relevance of LRG1 as a biomarker for early onset myocardial infarction.^19^ LRG1 was also the only biomarker found to be predictive of future HF, with non‐HF patients in the ‘high LRG1’ group found to be twice as likely to develop HF in the future. Overall, these data add weight to the suggestion that LRG1 could be a valuable biomarker for ventricular dysfunction and HF and may have particular utility in diagnosis of HFpEF.^8^ ZA2G was significantly elevated in HF patients, with a more significant association with HFrEF. Although generally researched in the context of cancer,^20^ it has previously been shown that serum levels of ZA2G are increased in HF patients.^21^ PON1 was also significantly differentially expressed between HF and non‐HF patients and significantly associated with HFrEF. Studies in two Japanese patient cohorts have revealed that the presence of certain alleles of PON1 increase the risk of carotid artery atherosclerotic disease^22^; however, the clinical significance of PON1 activity in cardiovascular conditions remains controversial. Some studies have associated low baseline PON1 activity with increased severity of coronary artery disease, while other studies report an association between high baseline PON1 activity and coronary artery disease severity.^23^ In a study by Hammadah *et al*, however, no correlation between HF events or hospitalizations and PON1 activity was observed.^23^ In this study, PON1 was down‐regulated in HF, although the more important observation regarding this protein was that, like ZA2G, it contributed strongly to the predictive performance of the combined biomarker model (*Table* *4*). Apolipoprotein I is significantly differentially expressed between both HF and non‐HF samples, even when adjusted for gender and AF, and had a more significant association with HFrEF. Apolipoproteins have been more strongly linked with HF and cardiovascular disease. Low APOA1 expression has been linked with more severe disease^24^ and conversely, higher levels of APOA1 are associated with reduced risk of major cardiovascular events.^25^ In our data, reductions in APOA1 were also observed in both HFpEF and HFrEF patients, when compared with non‐HF patients. The protein pentraxin 3 was also significantly elevated in serum from patients with HF; however, these changes were no longer significant when adjusted for AF. Individually, peptides for the proteins described above had poor predictive performance for HF (AUC 0.55–0.61). This finding again highlights the benefit of multiplexed measurement of biomarker combinations, rather than relying solely on the statistical significance of individual proteins for identification of clinically useful predictive biomarkers.

Although some of the candidate proteins did show potential to differentiate between HFpEF or HFrEF and non‐HF controls, the combined measurement of all candidate biomarker proteins did not have sufficient sensitivity or specificity to differentiate between either of the two disease subtypes (HFpEF and HFrEF). In this instance, it is likely that this is due to the low numbers of both HFpEF and HFrEF patients (*n* = 59 and *n* = 62, respectively) in relation to non‐HF controls (*n* = 289). To further elucidate the potential clinical utility of the biomarker panel, or selected candidate biomarkers within the panel, for specific diagnosis of HFpEF the biomarkers will have to be assessed in a more appropriately powered cohort of HFpEF patients and matched controls.

This study has some limitations. This Irish cohort is not representative of a diverse racial and ethnic population. Furthermore, all patients recruited, including non‐HF, represent an at‐risk population for future development of HF. Isoforms of BNP could not be included in the MRM method as there were limited options of proteotypic peptides for this protein and the only one that was deemed suitable was below the limits of detection. Indeed, 10 other biologically relevant candidate protein biomarkers were found to be below the limits of detection during assay development. These proteins, mainly the ‘known’ biomarkers, are routinely measured via immune assay‐based techniques. However, it is likely that due to their structure (short sequence length) and low abundance in serum, that these proteins will always prove difficult to measure via MRM unless alternative sample preparation and MRM methods are employed. Mass spectrometry technology is continually evolving, and it will be possible to further refine MRM assays for the low abundant proteins and peptides as part of on‐going work for development of the biomarker assay. Inclusion of these ‘known’ biomarkers in the MRM assay may further improve on the performance of the biomarker model described here. In addition, the cohort was not age matched, and age is a confounding risk factor in prediction of HF. However, when the samples were retrospectively matched based on age, the predictive performance of the combined biomarker model was not significantly impacted. Only a small number of non‐HF patients went on to develop HF, and so only exploratory survival analysis could be performed as part of this study. Definitive conclusions on the potential clinical utility of this combined biomarker panel for prediction of HF cannot be made until the panel is validated in an external cohort. This will include defining clinical thresholds for the complete biomarker panel, which will facilitate more accurate analysis of biomarker utility for prediction of future HF. These investigations will form part of a larger collaborative study, adhering to standard required for Clinical Laboratory Improvement Amendments‐accredited assays.

In order to be clinically useful for diagnosis and management of HF, candidate protein biomarkers should be easily measured in blood in a high‐throughput, cost‐effective, and reproducible manner in large sample numbers. Hence, there are a number of clinical tests now on offer, which avail of mass spectrometry to measure multiple proteins and/or protein isoforms in patient blood samples in short turn‐around times.^26^ The field of proteomics and mass spectrometry is developing rapidly, and advances have been made that will help bridge the gap between biomarker discovery, and development of a clinical test.^27^, ^28^ The costs of running an assay, such as what we have described here, has been estimated to be between €35 and €40 (£30–£35) per sample and turnaround time from sample receipt to provision of data would be 3–4 hours. In the United Kingdom, point of care BNP tests were previously estimated to cost an average of £25 per patient.^29^, ^30^ In Ireland, data from previous studiespatients and significantly associated withwithin the STOP‐HF cohort estimated the average cost of point‐of‐care BNP to be €20 per patient.^31^ Although more expensive than point‐of‐care BNP, it should be noted that the MRM assay described here is being developed to progress an evolving era in precision medicine, which BNP and other single markers cannot support. Therefore, the potential clinical value of such tests will justify the marginal increase in costs. This demonstrates the perceived clinical value in developing multimarker signatures such as what we have reported here. Thus, future work will be focused on further validating this biomarker panel in additional independent sample cohorts from different countries, which will require the establishment of clinical research collaborations in order to implement.

## Conflict of interest

None declared.

## Funding

This work was supported by Enterprise Ireland, the Health Research Board of Ireland, the British Heart Foundation, and Northern Ireland Chest Heart and Stroke (grant CF/2012/2607, CSA‐2012‐36, PG/17/91/33428, and NICHS/2019/10, respectively).

## Supporting information


**Table S1.** Candidate Biomarker Proteins.
**Table S3.** Correlation of Top Contributing Biomarkers with BNP.
**Figure S1.** Measurement of crude and synthetic peptides in serum.
**Figure S2.** Significantly Protein Expression Changes between non‐HF and HF patients.

**Table S2.** Multiple Reaction Monitoring (MRM) measurement data for all peptides in n = 406 patient samples. HF = heart failure; DHF ‐ diastolic heart failure (HFpEF); SHF = systolic heart failure (HFrEF).

**Table S3.** Spearman correlation coefficients between all measured peptides. Correlation coefficient greater than 0.8 indicate a strong colinear relationship (orange highlight). ‘Pep 1’ = peptide 1, ‘Pep 2’ = peptide 2.
