# Supplementary material for: Multiplexed measurement of candidate blood protein biomarkers of heart failure
Source: ESC Heart Fail. 2021 Mar 28;8(3):2248–58. doi: 10.1002/ehf2.13320 (PMC8120401; doi:10.1002/ehf2.13320)
Supplement: Supplementary file 1 — Table S1. Candidate Biomarker Proteins. Table S3. Correlation of Top Contributing Biomarkers with BNP. Figure S1. Measurement of crude and synthetic peptides in serum. Figure S2. Significantly Protein Expression Changes between non‐HF and HF patients. [file EHF2-8-2248-s003.docx]

**Supplementary Data**

**Supplementary Methods**

**Study Population**

A total of 500 patients were recruited for this study from the Heart Failure Unit and the Blood Pressure Unit at St. Michael’s Hospital, Dun Laoghaire, Dublin. All subjects gave written informed consent to participate in this study. The Ethics Committee at St. Vincent’s University Hospital approved the study protocol which conformed to the principles of the Helsinki Declaration. 150 heart failure (HF) patients were recruited; 75 heart failure patients with reduced ejection fraction (HFrEF) patients and 75 heart failure patients with preserved ejection fraction (HFpEF). All patients underwent clinical evaluation to identify study suitability. Heart failure status was defined using the New York Heart Association (NYHA) classification system. To be eligible for inclusion in the HF cohort, patients were required to have a previous hospitalisation for proven NYHA class IV HF (confirmed by an attending cardiologist), continued symptoms of at least NYHA class II HF. HFpEF patients had an ejection fraction ≥50% with Doppler echocardiography abnormalities of diastolic dysfunction (as defined by Lubien et al.)^1^ but no echocardiographic evidence of significant valvular heart disease. HFrEF patients had an ejection fraction <50%. A population of 350 non-heart failure (no-HF) patients were selected from the STOP-HF (St. Vincent’s Screening to Prevent Heart Failure) study, a pragmatic, prospective randomised trial in a broad community population which has recruited over 1400 patients who are over the age of 40 with at least one established risk factor for cardiac dysfunction, including: hypertension, hypercholesterolaemia, obesity, coronary artery disease or diabetes mellitus. These patients represent a high risk population for the future development of heart failure and served as a control group within this study.

**Patient Serum Collection**

Peripheral venous blood samples were obtained during clinical assessment. Serum samples were obtained following centrifugation at 2500 g for 10 min at 4^o^C. Samples were aliquoted and stored at -80ºC until required. Point of care BNP was measured using a Triage meter (Biosite). Assay sensitivity for BNP was 5 ng/mL. All steps, from collection to storage, were carried out at 4 ºC where possible and within a time-frame of <30 min. Each serum sample underwent no more than 3 freeze/thaw cycles prior to analysis. For assay development, a pooled serum sample was generated using three serum samples selected from the no-HF group, HFrEF group and the HFpEF group. This pooled sample was used as a reference for assay development.

**Sample Preparation for MRM Analysis**

Pooled patient serum samples were depleted in triplicate using Multiple Affinity Removal LC (MARS) [Hu-14 column, 4.6x100mm, Agilent Technologies], according to the manufacturer's instructions on a Biocad Vision Workstation. Fourteen high abundant serum proteins (albumin, transferrin, IgG, IgM, IgA, haptoglobulin, α-1 anti-trypsin, α-2 macroglobulin, α-1 glycoprotein, apolipoprotein A1, apolipoprotein A2, complement 3, transthyretin and fibrinogen), which account for 94% of the total protein mass were depleted. The remaining Low abundant protein (LAP) fraction was retained for LC-MRM/MS analysis. Verification of protein depletion was achieved via SDS PAGE gel electrophoresis analysis of crude and depleted serum sample. Crude and depleted serum samples were subjected to tryptic digestion. Briefly, 100 µg of crude and depleted Pooled serum sample were denatured [50% Trifluoroethanol, 10 mM DTT, 50 mM ammonium bicarbonate, 30 min, RT] in equal volumes using 5 kDa M spin filters (Sartorius Stedim Biotech). Samples were then alkylated with a final concentration of 20 mM iodacetamide (IAA) and incubated for a further 30 min in the dark. Denatured and alkylated serum samples were diluted 10 fold with a wash buffer [5% TFE, 50mM ammonium bicarbonate buffer] and subsequently concentrated in the 5 kDa Mw spin filters by centrifugation at 3000 g for 70 min at 4 ^o^C. The retained protein-containing serum samples were transferred to separate eppendorf tubes and digested with the addition of 0.2 µg/µl trypsin to achieve a protein:trypsin ratio of 1:50. Samples were then incubated in a thermomixer at 500 rpm for 18-24 hours at 37^o^C. Digested serum samples were purified using C18 resin ZipTips (R) (Millipore). Each tip contains C18 resin packed into a 10 µl pipette tip with a loading capacity of 5 µg protein per tip for the purification of proteins/peptides of molecular weight between 0-50 kDa. For purification of the peptides in the serum sample digests, 20 µl of sample was dried down under vacuum for approximately 30 min at 30 ^o^C and resuspended in 1 µg/µl 0.5% TFA. For peptide purification, the C18 resin was first 'wetted' with acetonitrile (x10). The resin was then equilibrated by pipetting 0.5% TFA (x10). Peptides were then bound to the pre-wetted C18 resin by pipetting the resuspended samples through the resin (x10). Bound peptides were eluted into fresh eppendorfs in 70% acetonitrile and 0.1% TFA. This process was repeated twice for each sample to ensure maximum peptide purification for MRM analysis. Purified samples were dried down under vacuum for approximately 30 min at 30 ^o^C and re-suspended in Buffer A [3% ACN, 0.1% formic acid] prior to MRM analysis.

**MRM Assay Design**

SwissProt accession numbers for each of the proteins were used to conduct a search for proteotypic peptides suitable for MRM analysis using Skyline (MacCoss laboratory, Washington DC version 1.4) and Spectrum Mill Peptide Selector (Agilent Technologies, version 3.3.078). Searches of the literature and previous in-house MRM data were also conducted. Peptides which adhered to the following criteria were selected; no missed cleavages in the peptide sequence, sequence length of 8-25 amino acids (AA), no potential ragged ends and no reactive cysteine (C) or methionine (M) residues within the sequence. Each peptide also had to match either an in house or public spectral library and qualify as being 'unique', as determined by the Skyline 'Uniqueness' search function. Where possible, peptides commonly identified in Skyline and Peptide Selector and/or recent literature were selected. In cases where there was no overlap, the highest ranking peptides identified by Skyline were chosen for development. In addition to the above criteria, the charge state of precursor ions was set to 2 or 3 and the product ions were limited to singly charged y and b-ions. In order to minimise potential interferences, ions with m/z close to the precursor ion were excluded. No fewer than 2 peptides were selected for each protein. Five transitions per peptide, with the highest MS signals in the available MS spectral libraries, were used for the initial MRM development. To aid in the development of MRM assays synthetic crude peptides were obtained from Thermo Fisher Scientific (PEPotecTM SRM unmodified peptide in plate). Prior to ordering, selected peptides were queried in the company's available software which provides feedback on peptide suitability for MRM analysis. Once received, synthetic peptides were pooled together to a final concentration of 200 pmol. The synthetic peptide pool was analysed in an Agilent 6460 Q-TOF mass spectrometer. Spectra required for MRM assay design in Skyline were built using the Trans Proteomic Pipeline software. Agilent .d files resulting from analysis of the peptide pool on the Q-TOF were first converted to mxXML format using MS Convert. Files were searched against a Uniprot Human database (Release 01-2011) using the X Tandem search engine to generate a combined pep.xml data file ready for import and spectral library building in Skyline. A probability score cut-off of 0.9 was applied to all spectral libraries generated in skyline. The spectral library data was used to build a transition list inclusive of the synthetic crude peptides, with five transitions per peptide as before. To determine the ability to detect target, low abundant peptides within a serum matrix via MRM, the synthetic peptide pool was spiked into serum samples at a ratio of 1:5 and analysed on a nanoflow reverse phase C18 chromatographic Chip Cube based separation coupled to an Agilent 6460 triple quadrupole mass spectrometer (QqQ).

**MRM Analysis**

MRM analysis was carried out on a nanoflow reverse phase C18 chromatographic Chip Cube based separation coupled to an Agilent 6460 triple quadrupole mass spectrometer (QqQ). One hundred µg of tryptically digested crude and depleted serum samples were reconstituted in 100 µl Buffer A [3% ACN 0.1% FA (H20)] and centrifuged for 30 min at 4 ^o^C. Samples were analysed using a 40 min separation LC run going from 0 - 95% Buffer B [10% H20 0.1% formic acid (ACN)] along the following gradient; [0 min 0% B, 5 min 10% B, 35 min 30% B, 37 min 95% B, 38 min 95% B 40 min 0% B]. Dwell time was set to 10 ms and collision energy was set with the Q-TOF equation for each transition [(precursor ion/100)*3.6-4.8]. The accelerator voltage was maintained at 4 V. For both crude and depleted serum, 2 µg of sample was loaded onto the HPLC chip at a flow rate of 3 µl/min. Following enrichment on a trapping column (160 nl), the peptides were separated on a C18 analytical column [Zorbax 3005B C18 5 µm (150 mm x 75 µm)] before being passed into the QqQ through a nanospray needle also contained in the chip. Prior to the start of the MRM runs, 5µl of PepMix was loaded under the same experimental conditions with an 18 min gradient [0 min 0% B, 9 min 35% B, 12 min 95% B, 15 min 95% B, 18 min 0% B] as a means of assessing system suitability. In order to prevent carry over between samples, 'blank' samples (3% ACN, 0.1% formic acid) were loaded at the start and end of each MRM run and between each sample injection. A stock peptide mix was used to confirm system suitability between each batch of samples analysed. A Buffer-only sample was analysed between individual samples to prevent sample carry over.

**Data analysis**

MRM data analysis was performed using both Qualitative Mass Hunter Software (Agilent, V 3.3.078) and Skyline (MacCoss lab, V 4.1). Qualitative Mass Hunter software was used for preliminary visual assessment of the results. Using this software, signal intensity based on the total ion chromatogram (TIC), was recorded for each measured peptide. A more automated analysis of the MRM analysis results was performed using Skyline software. This software provides information on dot product, ranking, retention time and correlation to spectral library data for each peptide and transition. Skyline software will automatically 'pick' what it believes to be the 'best peak' for a measured peptide. As such, it was necessary to manually review these selections in Skyline to ensure (based on DP and RT) that the true representative peak for each measured peptide was being analysed. IBM SPSS Statistics version 24 was used to process data and compare biomarker expression between groups. Non-parametric Mann-Whitney U and Kruskal-Wallis tests were used to investigate differences in biomarker expression between disease and control groups. Random Forest models were used to discriminate between the patient groups (Random Forest package in R V.4.3.2). The models were optimised to ensure that it did not overly focus on the majority group i.e. the non-HF group. This involved; (i) giving the random forest a heavier penalty for misclassifying a HF patient rather than a control and (ii) ensuring that each decision tree within the forest had a representative sample of both HF and control patients. Reported performance measures were cross-validated in all models using leave-one-out cross-validation. Measurements of accuracy (percentage of total cases correctly classified by the model) and the area under the receiver operating characteristic (ROC) curve (AUC), were calculated using the pROC package in R V.3.4.4.

**References**

[1] Lubien E, DeMaria A, Krishnaswamy P, Clopton P, Koon J, Kazanegra R, Gardetto N, Wanner E, Maisel AS. Utility of B-natriuretic peptide in detecting diastolic dysfunction: comparison with Doppler velocity recordings. Circulation United States; 2002;105:595–601.

**Supplementary Table 1. Candidate Biomarker Proteins**

|  | **Protein** | **Fold change between 'low' and 'high' risk patients^1^** | **Skyline** | **PS** | **Shared** | **IH** | **Literature** | **No. Peptides Evaluated** | **MRM Developed** |
| --- | --- | --- | --- | --- | --- | --- | --- | --- | --- |
| **Novel** | Complement factor I (CF1) | 6.8 increase | 2 | 1 | 1 | 0 | 0 | 2 | Y |
|  | Pigment epithelium-derived factor (PEDF) | 4.8 increase | 7 | 2 | 2 | 1 | 0 | 3 | Y |
|  | Fibronectin (FN1) | 4.8 increase | 2 | 16 | 0 | 0 | 0 | 2 | Y |
|  | Serum paraoxonase/arylesterase 1 (PON1) | 4.2 increase | 5 | 4 | 2 | 0 | 2 | 3 | Y |
|  | Beta-2-glycoprotein 1 (APOH) | 3.4 increase | 1 | 0 | 0 | 0 | 1 | 2 | Y |
|  | Apolipoprotein A-IV (APOA4) | 3.1 increase | 12 | 3 | 2 | 1 | 1 | 3 | Y |
|  | Apolipoprotein A-I (APOA1) | 2.6 increase | 17 | 2 | 1 | 2 | 1 | 3 | Y |
|  | Leucine-rich alpha-2-glycoprotein | 2.1 increase | 7 | 4 | 1 | 2 | 0 | 3 | Y |
|  | Complement C3 (C3) | 1.7 increase | 22 | 18 | 12 | 2 | 1 | 3 | Y |
|  | Zinc-alpha-2-glycoprotein | 1.7 increase | 9 | 3 | 3 | 1 | 0 | 4 | Y |
|  | Serum amyloid P-component (SAP) | 1.7 decrease | 6 | 2 | 2 | 2 | 0 | 3 | Y |
|  | Complement C1s subcomponent (C1S) | 1.7 decrease | 6 | 3 | 2 | 0 | 0 | 2 | Y |
|  | Vitamin D-binding protein (VTDB) | 1.6 decrease | 3 | 1 | 0 | 1 | 0 | 2 | Y |
|  | Gelsolin (GSN) | 1.6 decrease | 8 | 6 | 2 | 0 | 0 | 2 | Y |
|  | Transthyretin (TTHY) | 1.5 decrease | 1 | 0 | 0 | 0 | 0 | 1 | Y |
|  | Clusterin | 1.5 decrease | 8 | 3 | 2 | 2 | 0 | 3 | Y |
| **Known** | 72 kDa type IV collagenase (MMP2) | N/A | 6 | 2 | 0 | 0 | 0 | 3 | Y |
|  | Interleukin-6 | N/A | 1 | 3 | 1 | 0 | 0 | 1 | Y |
|  | Collagen alpha-2(I) chain | N/A | 5 | 2 | 1 | 0 | 0 | 3 | Y |
|  | Collagen alpha-1(III) chain | N/A | 0 | 1 | 0 | 0 | 0 | 1 | Y |
|  | Matrix metalloproteinase-9 (MMP9) | N/A | 0 | 4 | 0 | 0 | 0 | 1 | Y |
|  | Tissue inhibitor of metalloproteinase 1 (TIMP1) | N/A | 0 | 1 | 0 | 0 | 1 | 1 | Y |
|  | Galectin-3 | N/A | 3 | 0 | 0 | 0 | 0 | 3 | Y |
|  | Pentraxin-related protein PTX3 | N/A | 0 | 2 | 0 | 0 | 0 | 2 | Y |
|  | Macrophage migration inhibitory factor | N/A | 1 | 0 | 0 | 0 | 1 | 2 | Y |
|  | interleukin 8 | N/A | 0 | 0 | 0 | 0 | 0 | 0 | N |
|  | monocyte chemoattractant protein 1 (MCP1) | N/A | 0 | 3 | 0 | 0 | 0 | 2 | N |
|  | tumour necrosis factor α | N/A | 0 | 0 | 0 | 0 | 0 | 0 | N |
|  | Soluble ST2 | N/A | 0 | 0 | 0 | 0 | 0 | 0 | N |
|  | tissue inhibitor of metalloproteinase 2 (TIMP2) | N/A | 0 | 0 | 0 | 0 | 0 | 0 | N |
|  | carboxy-terminal propeptide of collagen 1 (PICP) | N/A | 0 | 10 | 0 | 0 | 0 | 3 | N |
|  | carboxy-terminal telopeptide of collagen 1 (CITP) | N/A | 1 | 3 | 0 | 0 | 0 | 1 | N |
|  | Natriuretic Peptide Receptor A (NPRA, GCA, NPR1) | N/A | 1 | 2 | 0 | 0 | 0 | 0 | N |
|  | BNP | N/A | 1 | 0 | 0 | 0 | 0 | 0 | N |

^1^Asymptomatic hypertensive patients stratified as ‘low’ and ‘high’ risk based in BNP levels. Y = yes; MRM = multiple reaction monitoring ; PS = Peptide Selector; Shared = identified in both Skyline and Peptide Selector; IH = In house MRM previously developed; Literature = peptides identified from previous publications

**Supplementary Table 3. Correlation of Top Contributing Biomarkers with BNP**

|  |  | **Zinc alpha 2 glycoprotein** | **Apolipoprotein AI** | | **Leucine rich 2 glycoprotein** | | **Transthyretin** | **Serum paraoxanase/arylesterase** | **Pigment epithelial derived factor** | **log(BNP)** |
| --- | --- | --- | --- | --- | --- | --- | --- | --- | --- | --- |
|  |  | **Peptide 1** | **Peptide 1** | **Peptide 2** | **Peptide 1** | **Peptide 2** | **Peptide 1** | **Peptide 1** | **Peptide 1** | **N/A** |
| **Zinc alpha 2 glycoprotein** | **Peptide 1** | 1 | .423** | .391** | .368** | .666** | .332** | .296** | .469** | .138** |
| **Apolipoprotein AI** | **Peptide 1** | .423** | 1 | .912** | .687** | .774** | .668** | .576** | .675** | -0.08 |
|  | **Peptide 2** | .391** | .912** | 1 | .684** | .714** | .792** | .554** | .647** | -0.077 |
| **Leucine rich 2 glycoprotein** | **Peptide 1** | .368** | .687** | .684** | 1 | .681** | .590** | .439** | .585** | 0.097 |
|  | **Peptide 2** | .666** | .774** | .714** | .681** | 1 | .515** | .520** | .691** | .115* |
| **Transthyretin** | **Peptide 1** | .332** | .668** | .792** | .590** | .515** | 1 | .557** | .611** | -0.061 |
| **Serum paraoxanase/arylesterase** | **Peptide 1** | .296** | .576** | .554** | .439** | .520** | .557** | 1 | .623** | -.123* |
| **Pigment epithelial derived factor** | **Peptide 1** | .469** | .675** | .647** | .585** | .691** | .611** | .623** | 1 | -0.068 |
| **log(BNP)** | **N/A** | **.138**** | **-0.08** | **-0.077** | **0.097** | **.115*** | **-0.061** | **-.123*** | **-0.068** | **1** |

*. Correlation is significant at the 0.01 level (2-tailed

**. Correlation is significant at the 0.05 level (2-tailed)


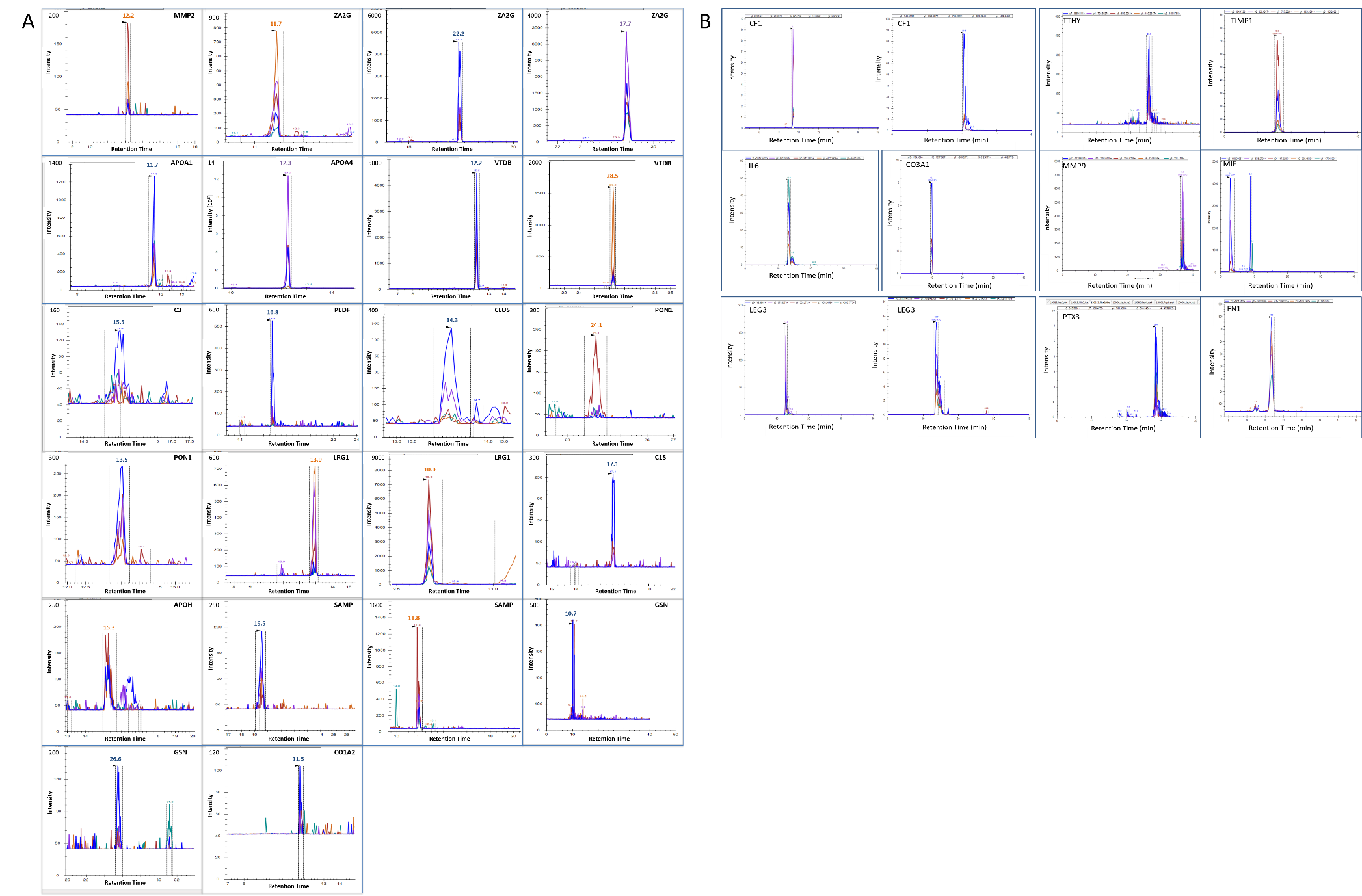


**Supplementary Figure 1: Measurement of crude and synthetic peptides in serum**

This figure shows the MRM data for the 22 endogenous peptides (15 proteins) detected in crude serum (A) and data for the synthetic peptides that were ‘spiked’ into crude serum samples (B), as viewed in Skyline. Signal intensity is recorded on the y-axis and retention time is recorded on the x-axis. Proteins are identified by their abbreviated names (*see Table 1*)

**Supplementary Figure 2. Significantly Protein Expression Changes between non-HF and HF patients.**

BNP expression is significantly different between non-HF and HF patients and BNP expression is normally distributed across HF patients. The difference in BNP expression between HFrEF and HFpEF is not significant when adjusted for atrial fibrillation. P-Values are adjusted for gender (blue) or Atrial Fibrillation (red) (A). Box plots of statistically significantly changing proteins (p≤0.05) between HF and non-HF patients (B) and HFpEF, HFrEF and non-HF patients (C) at the 95% confidence level. Unadjusted p-values are shown. Error bars reflect the 95% confidence interval. Non-HF = non-HF controls; HFrEF = HF with reduced ejection fraction; HFpEF = HF with preserved ejection fraction.
